# Mini-array of multiple tumor-associated antigens (TAAs) in the immunodiagnosis of breast cancer

**DOI:** 10.3892/ol.2012.1062

**Published:** 2012-12-05

**Authors:** HUA YE, CHANGQING SUN, PENGFEI REN, LIPING DAI, BO PENG, KAIJUAN WANG, WEI QIAN, JIANYING ZHANG

**Affiliations:** 1 Department of Epidemiology and Henan Key Laboratory of Tumor Epidemiology, College of Public Health, Zhengzhou University, Zhengzhou, Henan 450001, P.R. China;; 2 Department of Biological Sciences, the University of Texas at El Paso, El Paso, TX 79968, USA

**Keywords:** autoantibodies, tumor-associated antigens, immunodiagnosis, breast cancer

## Abstract

Sera from patients with cancer contain antibodies which react with a unique group of autologous cellular antigens called tumor-associated antigens (TAAs). This study aimed to determine whether a mini-array of multiple TAAs would enhance antibody detection and be a useful approach in breast cancer detection and diagnosis. The mini-array of multiple TAAs was composed of ten TAAs, including Imp1, p62, Koc, p53, c-myc, survivin, p16, cyclin B1, cyclin D1 and CDK2 full-length recombinant proteins. An enzyme-linked immunosorbent assay (ELISA) was used to detect antibodies against these ten TAAs in 41 sera from patients with breast cancer, as well as 82 sera from normal individuals. The antibody frequency of the individual TAAs in breast cancer was variable and ranged between 7.3 and 22.0%. With the successive addition of TAAs to a final total of ten antigens, there was a stepwise increase in positive antibody reactions, reaching a sensitivity of 61.0% and a specificity of 86.6% in breast cancer. The positive and negative likelihood ratios were 5.545 and 0.438, respectively, which showed that the clinical diagnostic value of a parallel assay of eight TAAs was high. The positive and negative predictive values were 73.5 and 82.0%, respectively, indicating that the parallel assay of eight TAAs raised the diagnostic precision significantly. The agreement rate and κ-value were 79.7% and 0.52, respectively, while the Youden’s Index (YI) was 0.5, indicating that the observed value of this assay had a middle range coincidence with the actual value. The data from the present study further support our previous hypothesis that the detection of autoantibodies for the diagnosis of certain types of cancer may be enhanced using a mini-array of several TAAs as target antigens. A customized antigen mini-array using a panel of appropriately selected TAAs is able to enhance autoantibody detection in the immunodiagnosis of breast cancer.

## Introduction

Breast cancer is the most frequent malignant tumor and a leading cause of cancer mortality among females in the majority of Asian countries, including China (1). Despite significant progress, 40% of patients diagnosed with breast cancer succumb to the disease. The high mortality rate may be attributed in part to a lack of diagnostic methods allowing early detection. Another major cause of mortality among breast cancer patients continues to be the presence of the metastatic disease with ∼5% of patients exhibiting clinically detectable metastases at the time of the initial diagnosis and a further 30–40% of patients with no clinically detectable disease harboring occult metastases. Although mammograms are the most effective tool for detecting breast cancer, the US Food and Drug Administration reports that mammography is able to identify only ∼80% of breast cancers in females (2). Hence, there is a requirement for further understanding of tumor biology and host response mechanisms so that new diagnostic and therapeutic tools may be developed. Early diagnosis is essential for the optimal management of breast cancer. Thus, extensive studies are being conducted to identify and validate new biomarkers to add to current markers and increase the sensitivity and specificity of breast cancer detection.

Numerous studies have demonstrated that cancer sera contain antibodies that react with a unique group of autologous cellular antigens called tumor-associated antigens (TAAs) (3,4). The types of cellular proteins that induce these autoantibody responses are varied and include tumor suppressors, such as p53 (5) and p16 (6), oncogene products, such as c-myc (7) and HER-2/neu (8), and other cancer-related proteins, such as Imp2/p62 (9), CRD-BP (10), CIP2A/p90 (11), survivin (12,13) and LEDGF (14). The various factors leading to the increased production of such autoantibodies are not completely understood. However, the available data show that a number of the target antigens are cellular proteins, such as p53, whose aberrant regulation or overexpression is capable of leading to tumorigenesis (5,15,16). The immune systems of certain cancer patients are able to sense these aberrant tumor-associated proteins as unknown antigens and have the capability to respond by producing autoantibodies (17). Thus, cancer-associated autoantibodies may be regarded as reporters identifying aberrant *de novo* or disregulated cellular mechanisms in tumorigenesis (3,4). The potential utility of TAA-autoantibody systems as early cancer biomarker tools to monitor therapeutic outcomes or as indicators of disease prognosis has been investigated. The present study evaluated whether a mini-array of multiple TAAs would enhance autoantibody detection and be an effective tool in the immunodiagnosis of breast cancer.

## Materials and methods

### Serum samples and antibodies

In the present study, sera from 41 patients with breast cancer and 82 normal individuals who had no clear evidence of malignancy were provided by our collaborator in China. Based on clinical information, all cancer sera were collected at the first time of diagnosis and patients did not receive any treatment with chemotherapy or radiotherapy. Normal control sera were collected during annual health examinations. The present study was approved by the Institutional Review Boards of the University of Texas at El Paso (UTEP) and collaborating academic institutions.

### Recombinant TAAs

All TAAs used in the present study, including Imp1, p62, Koc, p53, p16, c-myc, survivin, cyclin B1, cyclin D1, cyclin E and CDK2, were derived from our previous studies. The reactivities of these selected TAAs were determined with either polyclonal or monoclonal antibodies against the respective proteins.

### Enzyme-linked immunosorbent assay (ELISA)

Purified recombinant TAAs were individually diluted in PBS to a final concentration of 0.5 *μ*g/ml and 200 *μ*l were pipetted into each well to coat Immulon 2 microtiter plates (Fisher Scientific, Houston, TX, USA) overnight at 4°C. The human serum samples were diluted at 1:200, incubated with the antigen-coated wells at 37°C for 90 min followed by washing with PBS containing 0.05% Tween-20. The samples were then incubated with horseradish peroxidase (HRP)-conjugated goat anti-human IgG (Caltag Laboratories, Burlingame, CA, USA) as a secondary antibody diluted 1:2,000 for 90 min followed by washing with PBS containing 0.05% Tween-20. A solution of 3,3’,5,5’-tetramethyl benzidine (TMB)-H_2_O_2_-urea was used as the detecting agent. The OD of each well was read at 450 nm. Each sample was tested in duplicate. The cut-off value for determining a positive reaction was designated as the mean absorbance of the 82 normal human sera (NHS) plus 2 standard deviations (mean + 2SD). Since several hundred test sera were analyzed at various time periods, each run of the ELISA included at least 8 NHS samples and 2 positive control samples. These 8 NHS samples, representing a range of 2SD above and below the mean of the 82 NHS, were used in each experiment and the average value of the 8 NHS samples was used in each run to normalize all absorbance values to the mean of the entire 82 normal samples. In addition, all positive sera were confirmed with repeat testing, as were certain negative sera. The detailed protocol of the ELISA has been described previously (9,18).

### Western blotting and slot blot analysis

Western blot analysis was used to confirm that the bands observed in SDS-PAGE were reactive with the reference antibodies. In brief, the purified TAAs were electrophoresed by SDS-PAGE and subsequently transferred to a nitrocellulose membrane. The individual strips were pre-blocked in PBS containing 0.05% Tween-20 (PBST) with 5% non-fat milk for 30 min at room temperature, then incubated for 90 min with patient sera diluted 1:100 and finally incubated with HRP-conjugated goat anti-human IgG diluted 1:3,000 for 90 min, followed by washing with PBST solution. The positive signals were recorded by autoradiography. Slot blot analysis was used to confirm the positive sera samples detected by ELISA. The method for the slot blotting was identical to that of the western blotting with the exception that the purified recombinant protein (100 ng/well) was applied directly to the nitrocellulose membrane using a vacuum source. Membranes were not cut into strips and therefore, the detection of autoantibodies to all the TAAs in an individual patient’s serum was performed in one blot simultaneously.

### Statistical analysis

To determine whether the frequency of autoantibodies binding to selected TAAs in the cancer sera was significantly higher than that in NHS, the data were analyzed using the χ^2^ tests with Yates’ correction. Two levels of statistical significance (0.05 and 0.01) were used and P<0.05 was considered to indicate statistically significant differences. The comprehensive evaluations of the testing results for each anti-TAA antibody, including the methods for calculating the sensitivity, specificity, Youden’s index (YI), positive and negative likelihood ratio, positive (PPV) and negative predictive value (NPV), agreement rate and κ-value, were based on the methodology provided in the Epidemiology textbook (19).

## Results

### Prevalence of antibodies in a mini-array of multiple TAAs in breast cancer

**Figure 1.**
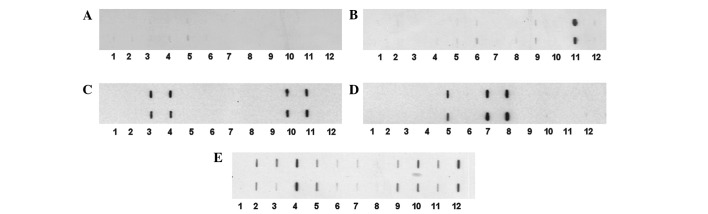
Mini-array of multiple TAAs with four representative breast cancer sera using slot blot analysis. Each blot represents a duplicate test for autoanti-bodies against a panel of eleven recombinant TAAs, with PBS as a negative control. Purified recombinant protein (100 ng per well) was applied directly to the nitrocellulose membrane using a vacuum device. Membranes were used for the simultaneous detection of autoantibodies in an individual patient’s serum to any of the eleven TAAs, following standard immunoblotting procedures. 1, PBS; 2, survivin; 3, p53; 4, p16; 5, cyclin B1; 6, cyclin D1; 7, cyclin E; 8, Koc; 9, Imp1; 10, p62; 11, CDK2; 12, c-myc. (A) Normal human serum showing no reactivity to any of the eleven TAAs. (B–E) Four representative breast cancer sera showing different antibody profiles with the 11 TAAs. TAA, tumor-associated antigen; PBS, phosphate-buffered saline.

In order to evaluate whether the combination of antibodies to multiple TAAs yields higher sensitivity for the diagnosis of breast cancer, the present study tested breast cancer sera for the presence of anti-TAA antibodies with a panel of ten selected recombinant TAAs using an ELISA and revealed that the combined the antibody frequency was 61.0% (25/41), significantly higher than the frequency (13.4%) in the sera from normal individuals (11/82). As shown in Table I, antibody frequency for any individual TAA in breast cancer was variable, ranging between 7.3 and 22.0%. The highest frequencies were against c-myc (22.0%), survivin (22.0%), cyclin B1 (17.1%) and cyclin D1 (17.1%), followed by p62 (12.2%), p53 (12.2%), p16 (12.2%), Imp1 (12.2%), CDK2 (9.8%) and Koc (7.3%). It was observed that, with the successive addition of TAAs to a total of eight antigens (c-myc, survivin, cyclin B1, cyclin D1, p62, p53, p12 and CDK2), there was a stepwise increase in the sensitivity, up to 61.0%, and the specificity was 89.0%. If additional antigens (Imp1 and Koc) were added to the panel, there was no further increase in the sensitivity (see Table II). These results indicate that an array of eight TAAs is able to serologically distinguish breast cancer patients from normal individuals at a sensitivity of 61.0%. However, it should be determined whether this TAA combination distinguishes breast cancer from other types of cancer. Positive results were also confirmed by slot blotting. Slot blot analysis of four representative breast cancer sera is shown in Fig. 1.

### Evaluation of diagnostic values of a mini-array of multiple TAAs in immunodiagnosis of breast cancer

The validity of a test is defined as its ability to distinguish between individuals who have a disease and those who do not. In order to address the question of how valuable the approach of antibody detection to a mini-array of multiple TAAs is in separating individuals with and without cancer, a group of parameters, including the sensitivity/specificity, YI and PPV/NPV, were calculated and are shown in Tables III and IV. Table III shows the comprehensive evaluation of antibodies to a panel of ten TAAs. With the successive addition of TAAs to a total of eight antigens, there was a stepwise increase in positive antibody reactions, up to 61.0%, as well as a slight decrease of specificity from 100% with one TAA to 89.0% with a panel of eight. If additional antigens (Imp1 and Koc) were added to the panel, there was no further increase in the sensitivity but a slight decrease of specificity from 89.0 to 86.6%. The sensitivity and specificity are consistent with the results of other two parameters (PPV/NPV). The PPV/NPVs were also variable in the various combinations of TAAs. In the panel with a total of eight TAAs, the PPV was 73.5% and the NPV was 82.0%. The YI was also increased from 0.220 with one TAA to 0.500 with eight TAAs. The positive and negative likelihood ratios were 5.545 and 0.438, respectively, indicating that the clinical diagnostic value of a parallel assay of five TAAs was high. This also suggests that a parallel assay of eight TAAs is able to raise the diagnostic accuracy significantly. The agreement rate and κ-value were 79.7% and 0.52, respectively, indicating that the observed value of this assay had a middle range coincidence with the actual value. Taken together, these data show the usefulness of the multiple antigen array in increasing the clinical diagnostic quality and value for cancer.

## Discussion

Interest in the use of anti-TAA antibodies as serological markers for cancer diagnosis derives from the recognition that these antibodies are generally absent, or present in very low titers, in normal individuals and in non-cancer conditions (with the exception of autoimmune conditions). The persistence and stability of autoantibodies in the serum of cancer patients is an advantage over other potential markers, including the TAAs themselves, which are released by tumors but are rapidly degraded or cleared after circulating in the serum for a limited time (17). Furthermore, the widespread availability of methods and reagents to detect serum autoantibodies facilitates their characterization in cancer patients and assay development. However, in contrast to autoimmune diseases, where the presence of a particular autoantibody may have diagnostic value, individually evaluated cancer-associated autoantibodies have little diagnostic value primarily due to their low frequency, sensitivity and specificity. This drawback may be overcome using mini-arrays of carefully selected TAAs and different types of cancer may require different TAA arrays to achieve the sensitivity and specificity required to make immunodiagnosis a feasible adjunct to tumor diagnosis (18,20–25).

In the future we aim to increase the sensitivity and specificity of anti-TAA antibodies as diagnostic markers of cancer by expanding the TAA array to include antigens which may be more selectively associated with one specific type of cancer, such as breast cancer, and not with others. We expect that our mini-array of multiple TAAs may be used as a novel non-invasive approach to identify cancer in the normal population and high-risk individuals. Our concern is that the approach may be not suitable for distinguishing one type of cancer from another. The reason is that certain TAAs, such as p53, p16 and c-myc, which were used in the present mini-array approach, are associated with several types of cancer, including liver, colon, gastric, lung, ovarian and prostate cancer (18,20,22–25). For future studies, we propose that certain selected antibody-antigen systems may be unique to one type of cancer and others may not. A comprehensive analysis and evaluation of various combinations of selected antibody-antigen systems is likely to be useful for the development of autoantibody profiles involving various panels or arrays of TAAs and the results may be useful for diagnosis of certain other types of cancer. In the present study, a mini-array of multiple TAAs were used as coating antigens in an ELISA to detect autoantibodies against these antigens in 41 sera from patients with breast cancer and 82 sera from normal individuals. The antibody frequency to the individual TAAs in breast cancer was variable and ranged between 7.3 and 22.0%. This relatively low sensitivity using one individual anti-TAA antibody as a diagnostic marker does not meet the requirements of clinical early diagnosis of breast cancer. With the successive addition of TAAs to a total of eight antigens, there was a stepwise increase in positive antibody reactions, reaching a sensitivity of 61.0% and a specificity of 89.0% in breast cancer. The positive and negative likelihood ratios were 5.545 and 0.438, respectively, which showed that the clinical diagnostic value of a parallel assay of eight TAAs was high. The PPVs and NPVs were 73.5 and 82.0%, respectively, indicating that the parallel assay of eight TAAs raised the diagnostic precision significantly. The agreement rate and κ-value were 79.7% and 0.52, respectively, which indicated that the observed value of this assay had a middle range coincidence with the actual value.

In conclusion, this preliminary study further supports our hypothesis and also suggests that additional breast cancer-specific TAAs are likely to be necessary to enhance the frequency of anti-TAA antibody detection using an array of multiple TAAs with potential immunodiagnostic value. Once a TAA array that is highly specific and sensitive to breast cancer is identified, we plan to develop a breast cancer-specific mini-array TAA chip for automated high-throughput breast cancer screening. Given that the presence of serum autoantibodies to TAAs may signal molecular events associated with tumorigenesis, it would be possible to use highly sensitive and specific TAA chips for screening populations at a high risk of developing breast cancer, which may lead to early preventive or therapeutic interventions aimed at suppressing or slowing the appearance of tumors.

## Figures and Tables

**Table I.** Frequency of antibodies for ten TAAs in breast cancer.

	No. (%) of autoantibodies in:	
Autoantibodies to:	BC (41)	NHS (82)
c-myc	9 (22.0)^b^	0 (0)
survivin	9 (22.0)^b^	1 (1.2)
cyclin B1	7 (17.1)^b^	1 (1.2)
cyclin D1	7 (17.1)^a^	2 (2.4)
p62	5 (12.2)^a^	1 (1.2)
p53	5 (12.2)^a^	2 (2.4)
p16	5 (12.2)^a^	2 (2.4)
Imp1	5 (12.2)^a^	2 (2.4)
CDK2	4 (9.8)^a^	1 (1.2)
Koc	3 (7.3)	1 (1.2)
Cumulative to ten antigens	61.0 (25/41)^b^	11 (13.4)

P-values relative to NHS,

a P<0.05,

b P<0.01. TAA, tumor-associated antigen; BC, breast cancer; NHS, normal human sera.

**Table II.** Sequential addition of antigen into the panel of ten TAAs in breast cancer.

	No. (%) of autoantibodies in:	
Antigen	BC (41)	NHS (82)
c-myc	9 (22.0)^a^	0 (0)
c-myc and survivin	14 (34.1)^a^	1 (1.2)
c-myc, surviving and cyclin B1	16 (39.0)^a^	2 (2.4)
c-myc, survivin, cyclin B1 and cyclin D1	18 (43.9)^a^	4 (4.9)
c-myc, survivin, cyclin B1, cyclin D1 and p62	20 (48.8)^a^	5 (6.1)
c-myc, survivin, cyclin B1, cyclin D1, p62 and p53	21 (51.2)^a^	7 (8.5)
c-myc, survivin, cyclin B1, cyclin D1, p62, p53 and p16	23 (56.1)^a^	9 (11.0)
c-myc, survivin, cyclin B1, cyclin D1, p62, p53, p16 and CDK2	25 (61.0)^a^	9 (11.0)
c-myc, survivin, cyclin B1, cyclin D1, p62, p53, p16, CDK2 and Imp1	25 (61.0)^a^	11 (13.4)
c-myc, survivin, cyclin B1, cyclin D1, p62, p53, p16, Imp1, CDK2 and Koc	25 (61.0)^a^	11 (13.4)

P-values relative to NHS:

a P<0.01. TAA, tumor-associated antigen; BC, breast cancer; NHS, normal human sera.

**Table III.** Evaluation of antibodies for ten TAAs selected in the detection of breast cancer.

	Positive % (No.)						
Panel of TAAs	BC (41)	NHS (82)	Sensitivity	Specificity	YI	PPV	NPV
c-myc	22.0 (9/41)^a^	0 (0/82)	22.0	100.0	0.220	100.0	71.9
c-myc+survivin	34.1 (14/41)^a^	1.2 (1/82)	34.1	98.8	0.329	93.3	75.0
c-myc+survivin+cyclin B1	39.0 (16/41)^a^	2.4 (2/82)	39.0	97.6	0.366	88.9	76.2
c-myc+survivin+cyclin B1+cyclin D1	43.9 (18/41)^a^	4.9 (4/82)	43.9	95.1	0.390	81.8	77.2
c-myc+survivin+cyclin B1+cyclin D1 +p62	48.8 (20/41)^a^	6.1 (5/82)	48.8	93.9	0.427	80.0	78.6
c-myc+survivin+cyclin B1+cyclin D1 +p62+p53	51.2 (21/41)^a^	8.5 (7/82)	51.2	91.5	0.427	75.0	78.9
c-myc+survivin+cyclin B1+cyclin D1 +p62+p53+p16	56.1 (23/41)^a^	11.0 (9/82)	56.1	89.0	0.451	71.9	80.2
c-myc+survivin+cyclin B1+cyclin D1 +p62+p53+p16+CDK2	61.0 (25/41)^a^	11.0 (9/82)	61.0	89.0	0.500	73.5	82.0
c-myc+survivin+cyclin B1+cyclin D1 +p62+p53+p16+CDK2+Imp1	61.0 (25/41)^a^	13.4 (11/82)	61.0	86.6	0.476	69.4	81.6
c-myc+survivin+cyclin B1+cyclin D1 +p62+p53+p16+Imp1+CDK2+koc	61.0 (25/41)^a^	13.4 (11/82)	61.0	86.6	0.476	69.4	81.6

P-values relative to NHS:

a P<0.01. TAA, tumor-associated antigen; BC, breast cancer; NHS, normal human sera; YI, Youden’s index; PPV, positive predictive value; NPV, negative predictive value.

**Table IV.** Summary of the diagnostic value of antibodies for a panel of eight TAAs in breast cancer.

Serum	Any TAA positive	All TAA negative	Total
BC	25 (A)	16 (C)	41 (R1)
NHS	9 (B)	73 (D)	82 (R2)
Total	34 (C1)	89 (C2)	123 (N)

Fourfold table χ^2^ tests: χ^2^=34.164, P=0.000. Sensitivity (%) = A/(A + C) = 25/41 = 61.0%. Specificity (%) = D/(B + D) = 73/82 = 89.0%. Youden’s index = Sensitivity + Specificity − 1 = 0.610 + 0.890 − 1 = 0.500. Positive (+) likelihood ratio = Sensitivity/(1 − Specificity) = 0.610/(1 − 0.890) = 5.545. Negative (-) likelihood ratio = (1 − Sensitivity)/Specificity = (1 − 0.610)/0.890 = 0.438. Percentage agreement = (A + D)/(A + B + C + D) × 100 = (25 + 73)/(25 + 16 + 9 +73) × 100 = 79.7%. κ = [N(A + D) − (R_1_C_1_ + R_2_C_2_)]/[N^2^ − (R_1_C_1_ + R_2_C_2_)] = 0.52. TAA, tumor-associated antigen; BC, breast cancer; NHS, normal human sera.
